# Supplementary material for: Complex‐centric proteome profiling by SEC‐SWATH‐MS
Source: Mol Syst Biol. 2019 Jan 14;15(1):e8438. doi: 10.15252/msb.20188438 (PMC6346213; doi:10.15252/msb.20188438)
Supplement: Supplementary file 7 — Dataset EV6 [file MSB-15-e8438-s007.zip › feature_plots_bioplex/Q9H0A8.pdf]

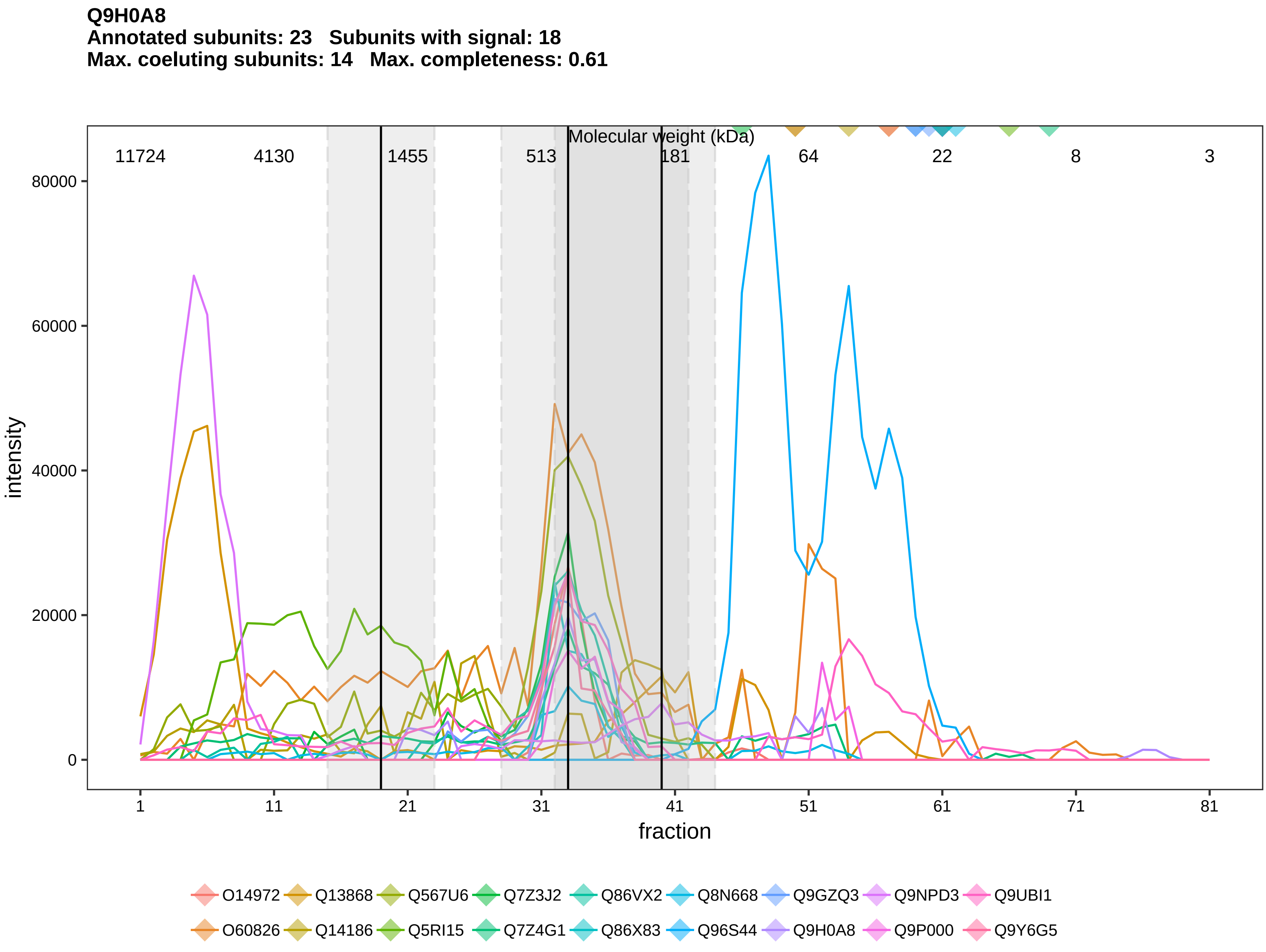
Q9H0A8
Annotated subunits: 23 Subunits with signal: 18
Max. coeluting subunits: 14 Max. completeness: 0.61
Molecular weight (kDa)
11724
4130
1455
513
181
64
22
8
3
intensity
80000
60000
40000
20000
0
1
11
21
31
41
51
61
71
81
fraction
O14972
Q13868
Q567U6
Q7Z3J2
Q86VX2
Q8N668
Q9GZQ3
Q9NPD3
Q9UBI1
O60826
Q14186
Q5RI15
Q7Z4G1
Q86X83
Q96S44
Q9H0A8
Q9P000
Q9Y6G5
